# Supplementary material for: Effectiveness and safety of acupuncture for postoperative ileus following gastrointestinal surgery: A systematic review and meta-analysis
Source: PLoS One. 2022 Jul 18;17(7):e0271580. doi: 10.1371/journal.pone.0271580 (PMC9292096; doi:10.1371/journal.pone.0271580)
Supplement: S1 Appendix — (DOCX) [file pone.0271580.s002.docx]

**S1 Appendix. Search strategy.**

| Embase  No. Query  #30 #9 AND #23 AND #29  #29 #24 OR #25 OR #26 OR #27 OR #28  #28 'randomly'  #27 'randomised'  #26 'randomized'  #25 'randomization'  #24 'random'  #23 #10 OR #22  #22 #11 OR #12 OR #13 OR #14 OR #15 OR #16 OR #17 OR #18 OR #19 OR #20 OR #21  #21 'fire needle': ab,kw,ti  #20 'triangle-edged needle':ab,kw,ti  #19 'abdominal acupuncture':ab,kw,ti  #18 'acupuncture points':ab,kw,ti  #17 'warm needling':ab,kw,ti  #16 'moxa needle':ab,kw,ti  #15 'transcutaneous electrical acupoint stimulation':ab,kw,ti  #14 'electroacupuncture':ab,kw,ti  #13 'manual acupuncture':ab,kw,ti  #12 'acupuncture therapy':ab,kw,ti  #11 'acupuncture':ab,kw,ti  #10 'acupuncture'/exp  #9 #1 OR #8  #8 #2 AND #7  #7 #3 OR #4 OR #5 OR #6  #6 'small bowel obstruction':ab,kw,ti  #5 'intestinal obstruction':ab,kw,ti  #4 'intestinal motility disorder':ab,kw,ti  #3 'intestinal dysfunction':ab,kw,ti  #2 'postoperative': ab,kw,ti  #1 'postoperative ileus'/exp  CNKI：  (SU=’术后肠梗阻’ OR SU=’术后肠功能障碍’ OR SU=’术后肠功能恢复’ ) AND (SU=’针灸疗法’ OR SU=’针灸’ OR SU=’针刺’ OR SU=’电针’ OR SU=’经皮穴位电刺激’ OR SU=’三棱针’ OR SU=’腹针’ OR SU=’梅花针’ OR SU=’火针’ OR SU=’温针’) AND (TKA=’随机’) |
| --- |
