# Supplementary material for: Effectiveness and safety of acupuncture for postoperative ileus following gastrointestinal surgery: A systematic review and meta-analysis
Source: PLoS One. 2022 Jul 18;17(7):e0271580. doi: 10.1371/journal.pone.0271580 (PMC9292096; doi:10.1371/journal.pone.0271580)
Supplement: S2 Appendix — (DOCX) [file pone.0271580.s003.docx]

**S2 Appendix. Sensitivity analysis.**

| Comparison | Outcome | Study omitted | Effect Estimate | Test for overall effect P-value | Heterogeneity | |
| --- | --- | --- | --- | --- | --- | --- |
|  |  |  |  |  | P | I^2^ (%) |
| Usual care | TFF | Xu SG 2020 | SMD -1.15 [-1.58, -0.72] | <0.00001 | < 0.00001 | 90 |
|  |  | Pu L 2019 | SMD -1.11 [-1.53, -0.69] | <0.00001 | < 0.00001 | 90 |
|  |  | Chen KB 2018 | SMD -1.18 [-1.60, -0.75] | <0.00001 | < 0.00001 | 90 |
|  |  | Kang H 2017 | SMD -1.13 [-1.57, -0.69] | <0.00001 | < 0.00001 | 90 |
|  |  | Jung SY 2017 | SMD -1.15 [-1.58, -0.73] | <0.00001 | < 0.00001 | 90 |
|  |  | Qian CL 2017 | SMD -1.07 [-1.47, -0.66] | <0.00001 | < 0.00001 | 89 |
|  |  | Xiao C 2014 (1) | SMD -1.11 [-1.54, -0.69] | <0.00001 | < 0.00001 | 90 |
|  |  | Xiao C 2014 (2) | SMD -1.17 [-1.60, -0.75] | <0.00001 | < 0.00001 | 90 |
|  |  | Tong 2014 | SMD -1.02 [-1.39, -0.65] | <0.00001 | < 0.00001 | 87 |
|  |  | Wang YK 2013 | SMD -1.09 [-1.50, -0.67] | <0.00001 | < 0.00001 | 89 |
|  |  | Ng S.S 2013 (2) | SMD -1.18 [-1.61, -0.74] | <0.00001 | < 0.00001 | 90 |
|  |  | Shi JE 2012 | SMD -1.06 [-1.46, -0.66] | 0.00001 | < 0.00001 | 89 |
|  |  | Yang JJ 2011 | SMD -1.18 [-1.60, -0.75] | <0.00001 | < 0.00001 | 90 |
|  |  | Wang HM 2011 | SMD -1.16 [-1.58, -0.74] | <0.00001 | < 0.00001 | 90 |
|  |  | Meng ZQ 2010 | SMD -1.22 [-1.62, -0.83] | <0.00001 | < 0.00001 | 88 |
|  |  | Garcia 2008 | SMD -1.22 [-1.62, -0.82] | <0.00001 | < 0.00001 | 89 |
|  |  | Combined | SMD -1.14 [-1.54, -0.73] | <0.00001 | < 0.00001 | 90 |
|  | TFD | Pu L 2019 | SMD -1.36 [-1.97, -0.74] | < 0.0001 | < 0.00001 | 94 |
|  |  | Kang H 2017 | SMD -1.30 [-1.94, -0.67] | <0.0001 | < 0.00001 | 94 |
|  |  | Jung SY 2017 | SMD -1.36 [-1.97, -0.76] | <0.00001 | < 0.00001 | 94 |
|  |  | Xiao C 2014 (1) | SMD -1.28 [-1.88, -0.67] | < 0.0001 | < 0.00001 | 94 |
|  |  | Xiao C 2014 (2) | SMD -1.37 [-1.98, -0.76] | < 0.0001 | < 0.00001 | 94 |
|  |  | Tong 2014 | SMD -1.02 [-1.46, -0.57] | < 0.00001 | < 0.00001 | 89 |
|  |  | Wang YK 2013 | SMD -1.22 [-1.79, -0.65] | < 0.0001 | < 0.00001 | 93 |
|  |  | Ng S.S 2013 (2) | SMD -1.36 [-1.99, -0.73] | < 0.0001 | < 0.00001 | 94 |
|  |  | Shi JE 2012 | SMD -1.22 [-1.81, -0.64] | <0.0001 | < 0.00001 | 94 |
|  |  | Yang JJ 2011 | SMD -1.35 [-1.96, -0.73] | < 0.0001 | < 0.00001 | 94 |
|  |  | Wang HM 2011 | SMD -1.37 [-1.97, -0.77] | < 0.00001 | < 0.00001 | 94 |
|  |  | Meng ZQ 2010 | SMD -1.42 [-2.00, -0.84] | < 0.00001 | < 0.00001 | 93 |
|  |  | Garcia 2008 | SMD -1.42 [-2.00, -0.84] | <0.00001 | < 0.00001 | 93 |
|  |  | Combined | SMD -1.31 [-1.88, -0.74] | <0.00001 | < 0.00001 | 94 |
|  | TBSR | Xu SG 2020 | SMD -1.63 [-2.25, -1.01] | <0.00001 | < 0.00001 | 92 |
|  |  | Pu L 2019 | SMD -1.59 [-2.22, -0.97] | < 0.00001 | < 0.00001 | 92 |
|  |  | Kang H 2017 | SMD -1.60 [-2.26, -0.94] | < 0.00001 | < 0.00001 | 92 |
|  |  | Qian CL2017 | SMD -1.44 [-2.00, -0.88] | < 0.00001 | < 0.00001 | 91 |
|  |  | XiaoC2014 (1) | SMD -1.59 [-2.21, -0.97] | < 0.00001 | < 0.00001 | 92 |
|  |  | XiaoC2014 (2) | SMD -1.63 [-2.25, -1.01] | < 0.00001 | < 0.00001 | 92 |
|  |  | Tong 2014 | SMD -1.24 [-1.62, -0.87] | < 0.00001 | < 0.00001 | 80 |
|  |  | Wang YK 2013 | SMD -1.58 [-2.23, -0.93] | < 0.00001 | < 0.00001 | 92 |
|  |  | Shi JE2012 | SMD -1.63 [-2.25, -1.01] | < 0.00001 | < 0.00001 | 92 |
|  |  | Yang JJ 2011 | SMD -1.68 [-2.28, -1.08] | < 0.00001 | < 0.00001 | 92 |
|  |  | Wang HM2011 | SMD -1.72 [-2.29, -1.15] | < 0.00001 | < 0.00001 | 91 |
|  |  | Combined | SMD -1.57 [-2.14, -1.01] | < 0.00001 | < 0.00001 | 91 |
|  | LOS | Xu SG 2020 | MD -1.55 [-2.63, -0.47] | = 0.005 | < 0.00001 | 87 |
|  |  | Pu L 2019 | MD -1.93 [-2.80, -1.05] | < 0.0001 | < 0.00001 | 82 |
|  |  | Chen KB2018 | MD -1.72 [-2.70, -0.75] | = 0.0005 | < 0.00001 | 88 |
|  |  | Kang H 2017 | MD -1.28 [-2.02, -0.54] | = 0.0007 | = 0.0001 | 78 |
|  |  | Jung SY2017 | MD -1.83 [-2.76, -0.90] | = 0.0001 | < 0.00001 | 87 |
|  |  | Qian CL 2017 | MD -1.60 [-2.84, -0.36] | = 0.01 | < 0.00001 | 88 |
|  |  | Garcia 2008 | MD -1.91 [-2.79, -1.03] | < 0.0001 | < 0.00001 | 86 |
|  |  | Ng SS 2013 (2) | MD -1.63 [-2.60, -0.66] | = 0.001 | < 0.00001 | 88 |
|  |  | Combined | MD -1.68 [-2.55, -0.80] | 0.0002 | < 0.00001 | 86 |
| Sham | TFF | Gu SH 2019 | SMD -0.88 [-1.81, 0.05] | = 0.06 | = 0.0001 | 89 |
|  |  | Yuan L 2017 | SMD -0.50 [-0.75, -0.25] | < 0.0001 | = 0.36 | 3 |
|  |  | Zhang ZD 2014 | SMD -0.90 [-1.66, -0.14] | = 0.02 | = 0.0001 | 89 |
|  |  | Ng S.S 2013 (1) | SMD -1.01 [-1.76, -0.26] | = 0.008 | = 0.003 | 83 |
|  |  | Combined | SMD -0.81 [-1.40, -0.23] | = 0.007 | = 0.0004 | 83 |
|  | TFD | Gu SH 2019 | SMD -0.40 [-0.73, -0.07] | = 0.07 | = 0.24 | 27 |
|  |  | Zhang ZD 2014 | SMD -0.39 [-0.65, -0.12] | = 0.004 | = 0.35 | 0 |
|  |  | Ng S.S 2013 (1) | SMD -0.22 [-0.53, 0.10] | = 0.17 | = 0.62 | 0 |
|  |  | Combined | SMD -0.34 [-0.58, -0.10] | = 0.006 | = 0.44 | 0 |
|  | TBSR | Gu SH 2019 | SMD -1.10 [-2.26, 0.06] | = 0.06 | = 0.008 | 86 |
|  |  | Yuan L 2017 | SMD -0.79 [-1.15, -0.43] | < 0.0001 | = 0.29 | 12 |
|  |  | Zhang ZD 2014 | SMD -1.26 [-2.02, -0.50] | = 0.001 | = 0.03 | 79 |
|  |  | Combined | SMD -1.03 [-1.64, -0.43] | = 0.0008 | = 0.02 | 74 |
|  | LOS | Yuan L 2017 | MD -0.39 [-1.08, 0.29] | = 0.26 | = 0.50 | 0 |
|  |  | Zhang ZD 2014 | MD -1.01 [-2.47, 0.46] | = 0.18 | = 0.02 | 82 |
|  |  | Ng SS 2013 (1) | MD -1.62 [-2.51, -0.72] | = 0.0004 | = 0.46 | 0 |
|  |  | Combined | MD -0.99 [-2.06, 0.08] | = 0.07 | = 0.06 | 64 |

Abbreviations: SMD, standard mean difference; MD, standard mean difference; TFF, time to first flatus; TFD, time to first defecation; TBSR, time to bowel sounds recovery; LOS, length of hospital stay.
