# Supplementary material for: Effectiveness and safety of acupuncture for postoperative ileus following gastrointestinal surgery: A systematic review and meta-analysis
Source: PLoS One. 2022 Jul 18;17(7):e0271580. doi: 10.1371/journal.pone.0271580 (PMC9292096; doi:10.1371/journal.pone.0271580)
Supplement: Attachment — Submitted filename: Rebuttal Letter.docx [file pone.0271580.s004.docx]

**Dear Editor:**

Thank you for arranging a timely review for our manuscript entitled “Effectiveness and safety of acupuncture for postoperative ileus following gastrointestinal surgery: A systematic review and meta-analysis” (PONE-D-22-01716). All the comments are valuable and helpful for improving our paper, as well as the important guiding significance to our researches. We have carefully read the reviewers’ critical comments and thoughtful suggestions, and revised the manuscript by responding to these suggestions point-by-point. We revised the manuscript and marked all changes in yellow. Below, we have answered the reviewers’ comments and suggestions point by point:

**Responds to the reviewer’s comments:**

**Reviewer #1**:

1. **Comment**: The authors describe their methodology in detail and outline the inclusion criteria. Please could the authors state why only RCTs were included and non-randomised trials were not considered for inclusion.

***Answer:*** Thank you for your comment. RCTs are considered the gold standard of evidence-based medicine for health interventions because they are designed to minimize the risk of bias. The non-randomised trials (NRSI) could not satisfactorily eliminate possible biases due to other factors (apart from treatment), which results may be affected by other confounding factors and may severely compromise the validity of their results [*Altman DG, Bland JM. Statistics notes. Treatment allocation in controlled trials: why randomise? BMJ. 1999, 318(7192):1209.*]. Randomized design can balance potential confounding factors and ensure the homogeneity of the two groups of patients. Therefore, evidence in RCT studies has high internal consistency and can better reflect the net effect of acupuncture therapies. Furthermore, there is no difference on average in the risk estimate of adverse effects of an intervention derived from meta-analyses of RCTs and meta-analyses of observational studies [*Golder S, Loke YK, Bland M. Meta-analyses of adverse effects data derived from randomised controlled trials as compared to observational studies: methodological overview. PLoS Med. 2011, 8(5):e1001026.*]. Since the purpose of the present study was to assess the effectiveness and safety of acupuncture for postoperative ileus following gastrointestinal surgery, we considered only RCTs for inclusion in this review.

We also noticed that the applicability of RCT results is limited due to restrictive selection criteria. In contrast, NRSI are generally more likely to reflect real-life clinical practice because they have a wider range of participants, longer follow-up time. Incorporate data from NRSIs to complement RCTs can generate more comprehensive evidence to guide healthcare decisions [1. *Faber T, Ravaud P, Riveros C, Perrodeau E, Dechartres A. Meta-analyses including non-randomized studies of therapeutic interventions: a methodological review. BMC Med Res Methodol. 2016, 22;16:35.* 2*. Sarri G, Patorno E, Yuan H, Guo JJ, Bennett D, Wen X, Zullo AR, Largent J, Panaccio M, Gokhale M, Moga DC, Ali MS, Debray TPA. Framework for the synthesis of non-randomised studies and randomised controlled trials: a guidance on conducting a systematic review and meta-analysis for healthcare decision making. BMJ Evid Based Med. 2022, 27(2):109-119.*]. This limitation and recommendation have been strengthened and pointed out in revised manuscript. After a systematic review of the evidence from the RCTs, we will review the data from NRSIs from a more pragmatical perspective in the future.

2. **Comment**: The methods also describe the search strategy, please could the authors state if expert support was sought to define the search strategy, such as from an information specialist.

***Answer***: Yes. Our work is a part of the project funded by Shanghai Clinical Research Center for Acupuncture and Moxibustion (No. 20MC1920500) and National Administration of Traditional Chinese Medicine: 2019 Project of building evidence based practice capacity for TCM (No. 2019XZZX-ZJ0011). Therefore, the search strategy of this work was guided by the expert group of Shanghai Clinical Research Center for Acupuncture and Moxibustion and China Center for Evidence Based Traditional Chinese Medicine--Acupuncture and Moxibustion Advantageous Diseases Project Team, which included information specialist.

3. **Comment**: The authors correctly investigate risk of bias across all studies. Several RCTs included are reported to have high risk of bias components or unclear risk of bias. Please could the authors further define how this was accounted for in the meta-analysis.

***Answer***: Thank you for your rigorous consideration. According to your suggestion, we have added assessment details in the Result section and judgements about the risk of bias affecting the quality of evidence in the discussion section to clarify this point.

Risk-of-bias assessment is a central component of systematic reviews. We assessed the methodological qualities of all studies in strict accordance with the Cochrane handbook [*Higgins JPT, Altman DG, Sterne JAC (editors). Chapter 8: Assessing risk of bias in included studies. In: Higgins JPT, Churchill R, Chandler J, Cumpston MS (editors), Cochrane Handbook for Systematic Reviews of Interventions version 5.2.0 (updated June 2017), Cochrane, 2017*.]. In Cochrane handbook, the criteria for the judgement of ‘high risk’ of bias in this domain was: no blinding or incomplete blinding, and the outcome was likely to be influenced by lack of blinding; blinding of key study participants and personnel attempted, but likely that the blinding could have been broken, and the outcome was likely to be influenced by lack of blinding. In this study, we found that some trials didn’t use blind method so we judged them as ‘high risk’. The criteria for the judgement of ‘unclear risk’ of bias in the domain of allocation concealment was: Insufficient information available to permit a judgement of ‘low risk’ or ‘high risk’ which usually the case if the method of concealment is not described or not described in sufficient detail to allow a definite judgement – for example if the use of assignment envelopes was described, but it remains unclear whether envelopes were sequentially numbered, opaque and sealed. In this study, there were thirteen trials didn’t report the details of the allocation concealment so that we permit a judgment of ‘unclear risk’. The criteria for the judgement of ‘unclear risk’ of bias in the domain of blinding of outcome assessment was: insufficient information available to permit a judgement of ‘low risk’ or ‘high risk’; the study did not address this outcome. Fifteen included trials in this meta-analysis didn’t adequately descripted whether the outcome assessors were blinded to the treatment allocation so were judged as ‘unclear risk’.

The results of the assessment showed that the high risk of bias mainly existed in the domain of ‘blinding of participants and personnel’, and the unclear risk mainly existed in the domain of ‘allocation concealment’ and ‘blinding of outcome assessment’. Serious risk of bias was the main reason for rating down the quality of evidence. The lack of blinding and uncertain in allocation concealment may lead to an overestimation or underestimation of the true effects of intervention *[Reveiz L, Chapman E, Asial S, Munoz S, Bonfill X, Alonso-Coello P. Risk of bias of randomized trials over time. J Clin Epidemiol. 2015; 68(9):1036–45.*]. However, it should be noted that blinding of participants and practitioners is difficult due to the nature of acupuncture intervention. But as far as possible the participants should be blinded whenever possible to ensure that participants cannot distinguish the real acupuncture from a sham control. In addition, the blinding of outcome assessors and statisticians is necessary and the specific process should be reported.

**Reviewer #2**:

1. **Comment**: In my opinion this article lacks of explanation of what really means usual care in this particular clinical condition. There is only an hint in the “Introduction” (lines 76-78) of potential treatments to reduce the occurrence of POI

***Answer***: Thank you for your suggestion**.** We have added definitions of usual care to the introduction section. The usual care patient received after GI surgery mainly includes routine nasogastric tubes, intravenous fluids, parenteral nutrition and early mobilization [*Wattchow D, Heitmann P, Smolilo D, Spencer NJ, Parker D, Hibberd T, Brookes SSJ, Dinning PG, Costa M. Postoperative ileus-An ongoing conundrum. Neurogastroenterol Motil. 2021 May;33(5):e14046.*]. Meanwhile, according to reviewer #3’s suggestion, we also supplemented a brief information of usual care in each included RCT in the Table 1.

2. **Comment**: In the paragraph “Acupoints combination” are mentioned two of the acupuncture’s basic method to treat, the combination of distal and proximal acupoints, but it’s not well explained what does it mean (does proximal mean closer to the gastro-intestinal district?) I think it’s important and more complete to point out the difference.

***Answer***: We are very sorry we didn't make it clear, your understanding is correct. We have added definitions to the discussion of “Acupoints combination” section.

3. **Comment**: The authors mention the prevalence of the use of ST36, but this is not explained why. ST 36 is one of the master point in Traditional Chinese Medicine, due to its efficacy and tropism for all gastro-intestinal disorders, that’s the reason why is so largely used.

***Answer***: Thank you for your important suggestion. We have added this point into the discussion of “Acupoints combination” section. Thanks again.

**Reviewer #3:**

1. **Comment:** It proposed that the Table 1 should report more details, including course of treatment and a brief information of usual care in the control group.

***Answer*:** Thank you for the valuable suggestions. We have supplement details of the course of treatment and brief information of usual care in Table 1.

2. **Comment**: For outcomes time to first flatus, time to first defecation and time to bowel sounds recovery, SMD was used as an effect size to synthesize data, which does not contribute to the understanding of the clinical importance of the difference. What is the rationale?

***Answer:*** Thank you for pointing out an important question. The mean difference (MD) is the difference in the means of the treatment group and the control group, while the standardized mean difference (SMD) is the MD divided by the standard deviation (SD), derived from either or both of the groups. As you said, the overall treatment effect in terms of SMD can be difficult to interpret as it is reported in units of standard deviation rather than in units of any of the measurement scales used in review [*Egger M, Smith GD, Altman D. Systematic Reviews in Health Care: Meta-Analysis in Context. 2008. Wiley. com.*]. However, the MD from different RCTs with different units cannot be pooled in meta-analysis to yield a summary estimate. *Part 2, Chapter 9, of the Cochrane Handbook* pointed out that the choice between MD and SMD depends on whether “outcome measurements in all studies are made on the same scale.” [*Higgins JP, Green S. Cochrane Handbook for Systematic Reviews of Interventions, Version 52.0 [Updated June 2017]. London, UK: The Cochrane Collaboration; 2017*]. One study suggested that the SMD does not depend on the unit of measurement, and therefore the SMD has been widely used as a measure of intervention effect in many applied fields [*Tian L. Inferences on standardized mean difference: the generalized variable approach. Stat Med. 2007 Feb 28;26(5):945-53.*]. The use of SMD in this study is based on the following considerations: First, the time to first flatus, time to first defecation and time to bowel sounds recovery are three important evaluation indicators to measure the clinical efficacy of acupuncture for POI following GI surgery. The units of measurement for these outcomes are different across the included studies, some are measured in days, some are measured in hours. Second, Although the changes of SMD couldn’t directly reflect the clinical importance of the difference since it doesn’t have units, we can use the Cohen’s *d* as the measure of effect size to show the significance of difference between two groups [*Andrade C. Mean Difference, Standardized Mean Difference (SMD)* and Their Use in Meta-Analysis: As Simple as It Gets*.* *J Clin Psychiatry. 2020 Sep 22;81(5):20f13681.*]. It is widely accepted that SMD values of 0.2-0.5 are considered a “small” effect, values of 0.5-0.8 are considered a “medium” effect, and values > 0.8 are considered a “large” effect [*Patrick Schober. Statistics From A (Agreement) to Z (z Score): A Guide to Interpreting Common Measures of Association, Agreement, Diagnostic Accuracy, Effect Size, Heterogeneity, and Reliability in Medical Research; Cohen J. The t test for means. In: Statistical Power Analysis for the Behavioral Sciences. Psychology Press, Taylor & Francis Group, 1988:19–74.*]. Third, studies for which the difference in means is the same proportion of the standard deviation (SD) will have the same SMD, regardless of the actual scales used to make the measurements [*Cochrane Handbook version 5.2.0 Chapter 6.5.1.2*]. So the SMD be likewise concerned as MD because this means that the curve representing the distribution has been shifted to the right by the same whole SD [*Andrade C. Mean Difference, Standardized Mean Difference (SMD)* and Their Use in Meta-Analysis: As Simple as It Gets*.* *J Clin Psychiatry. 2020 Sep 22;81(5):20f13681.*]. Forth, a study, which included 1068 meta-analyses, found that there were no differences in the percentage of statistical significance between MD and SMD in either model [*Takeshima N, Sozu T, Tajika A, Ogawa Y, Hayasaka Y, Furukawa TA. Which is more generalizable, powerful and interpretable in meta-analyses, mean difference or standardized mean difference? BMC Med Res Methodol. 2014 Feb 21;14:30.*]. Therefore, we use SMD to combine the outcomes in the meta-analyses. We have added text and clarification in the revised manuscript.

3. **Comment:** It suggests adding the description for any methods used to assess certainty in the body of evidence for some outcomes and their results.

***Answer:*** We appreciate the reviewer’s helpful suggestion. In the revised manuscript, we have added the description of GRADE approach which assessed the level of certainty in the evidence for intervention to the Method section. In addition, we also supplement the summary of certainty assessment results to the Results section and discussed the quality of evidence.
