# Supplementary material for: Effectiveness and safety of acupuncture for postoperative ileus following gastrointestinal surgery: A systematic review and meta-analysis
Source: PLoS One. 2022 Jul 18;17(7):e0271580. doi: 10.1371/journal.pone.0271580 (PMC9292096; doi:10.1371/journal.pone.0271580)
Supplement: Attachment — Submitted filename: Rebuttal Letter-R2.docx [file pone.0271580.s005.docx]

**Dear Editor:**

Thank you for arranging a timely review for our manuscript entitled “Effectiveness and safety of acupuncture for postoperative ileus following gastrointestinal surgery: A systematic review and meta-analysis” (PONE-D-22-01716R1).

The manuscript has been polished by an English language editing company (<https://www.cwauthors.com/>). We revised the manuscript and marked all changes in yellow. We also reviewed reference list and ensured that they were complete and correct. The cited papers were not retracted.
